# Sinus Node Dysfunction in Takotsubo Syndrome: An Uncommon Presentation

**DOI:** 10.1016/j.jaccas.2026.108153

**Published:** 2026-05-05

**Authors:** Georgios E. Zakynthinos, Konstantinos Kalogeras, Georgios J. Vlachojannis, George Makavos, Nikolaos K. Kokkinos, Nektarios Souvaliotis, Efstratios Katsianos, Nikolaos Vythoulkas-Biotis, Polychronis Dilaveris, Gerasimos Siasos

**Affiliations:** Third Department of Cardiology, “Sotiria” Chest Diseases Hospital, Medical School, National and Kapodistrian University of Athens, Athens, Greece

**Keywords:** cardiac magnetic resonance, left ventricle, shortness of breath, supraventricular arrhythmias, systolic heart failure

## Abstract

**Background:**

Stress cardiomyopathy is an acute, usually reversible left ventricular (LV) systolic dysfunction often triggered by emotional or physical stress. Conduction system complications are uncommon.

**Case Summary:**

A 68-year-old patient presented with acute pulmonary edema, reduced LV ejection fraction, and troponin elevation. Coronary angiography excluded obstructive coronary artery disease, and cardiac magnetic resonance confirmed stress cardiomyopathy. During hospitalization, the patient developed sinus node dysfunction (SND). Despite complete recovery of LV systolic function, the conduction abnormality persisted, requiring permanent pacemaker implantation.

**Discussion:**

Arrhythmias occur in up to one-quarter of patients with stress cardiomyopathy, most commonly atrial fibrillation or ventricular arrhythmias, whereas persistent SND is rare. This case highlights that recovery of ventricular systolic function does not necessarily indicate recovery of conduction abnormalities and underscores the need for careful rhythm monitoring and individualized pacing decisions.

**Take-Home Message:**

Persistent SND may complicate stress cardiomyopathy and may require permanent pacemaker implantation.

## Visual Summary

**Figure:**
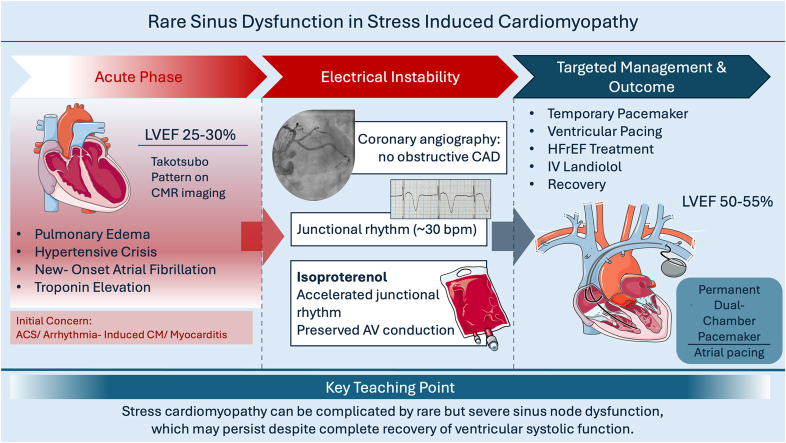

**Visual Summary:**
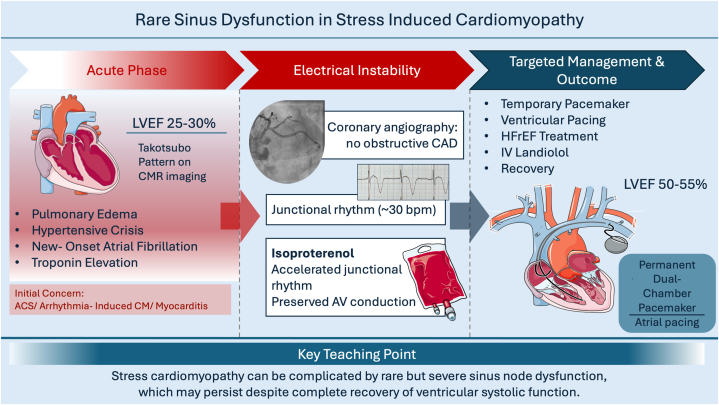
Clinical Course of Stress Cardiomyopathy Complicated by Rare Sinus Node Dysfunction The patient presented with acute heart failure, atrial fibrillation, and reduced left ventricular systolic function. Electrocardiography revealed marked repolarization abnormalities and profound sinus node dysfunction. Cardiac magnetic resonance confirmed stress cardiomyopathy. Despite improvement of repolarization abnormalities and recovery of left ventricular systolic function, sinus node function did not recover, leading to permanent dual-chamber pacemaker implantation with atrial pacing. Image(s) provided by Servier Medical Art,^11^ licensed under CC BY 4.0.^12^ ACS = acute coronary syndrome; AV = atrioventricular; CAD = coronary artery disease; CM = cardiomyopathy; CMR = cardiac magnetic resonance; HFrEF = heart failure with reduced ejection fraction; IV = intravenous; LVEF = left ventricular ejection fraction.

## History of Presentation

A 68-year-old man presented to the emergency department with progressive acute dyspnea over the last 24 hours, with a 60-minute episode of chest pain. On admission, he was hypertensive, tachypneic, and hypoxemic, with clinical signs of acute pulmonary edema. Electrocardiography demonstrated atrial fibrillation with rapid ventricular response, along with mild T-wave negativity in leads V_2_-V_6_ and subtle ST-segment elevation without an ischemic pattern; the QT interval was within normal limits.

**Take-Home Message**

• Persistent sinus node dysfunction even after recovery of left ventricular systolic function is a rare complication of stress cardiomyopathy, which might necessitate permanent pacemaker implantation.

Laboratory evaluation revealed markedly elevated cardiac troponin I levels on admission (2,235 pg/mL; upper reference limit 34 pg/mL), with a peak value of 2,520 pg/mL on the following day, as well as elevated B-type natriuretic peptide levels (2,950 pg/mL). Renal function was impaired at presentation, with a serum creatinine level of 2.0 mg/dL. Point-of-care transthoracic echocardiography demonstrated newly reduced left ventricular (LV) systolic function. The patient was subsequently admitted to the coronary care unit for further management and diagnostic evaluation.

## Past Medical History

The past medical history was notable for untreated arterial hypertension. The patient had no history of smoking, no family history of cardiomyopathy or sudden cardiac death, and was not receiving any chronic medications.

## Differential Diagnosis

The differential diagnosis included acute coronary syndrome, arrhythmia-induced cardiomyopathy (AIC), acute myocarditis, and stress cardiomyopathy (Takotsubo syndrome [TTS]).

## Investigations

Initial electrocardiography showed a newly diagnosed atrial fibrillation with rapid ventricular response. Transthoracic echocardiography showed diffuse LV hypokinesis, predominant in the mid-apical segments, with severely reduced ejection fraction (30%, Simpson biplane). No clear apical ballooning was visualized, likely due to suboptimal acoustic windows. Right ventricular function was normal (Video 1).

**Figure 1:**
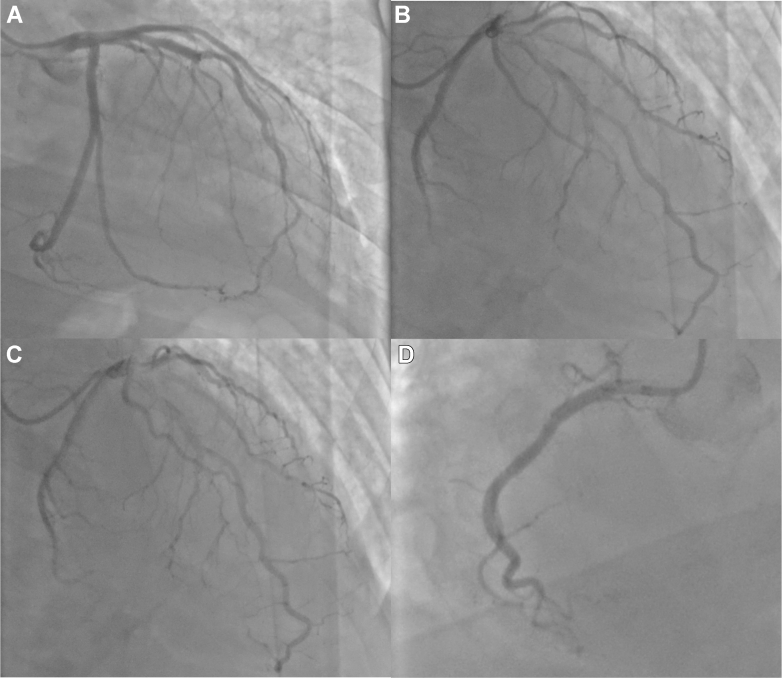
Coronary Angiography Coronary angiography demonstrating a left-dominant coronary system. (A to C) The left coronary circulation without angiographically significant obstructive disease. (D) A small, nondominant right coronary artery, also without obstructive lesions.

Given the acute onset of dyspnea, troponin elevation, electrocardiogram abnormalities, and reduced ejection fraction, urgent coronary angiography was performed within the first 24 hours and revealed no obstructive epicardial coronary artery disease (Figure 1).

During the first hospital day, the patient developed profound bradycardia with junctional rhythm (approximately 30 beats/min), marked QT prolongation (620 milliseconds), and deep T-wave inversions. These findings were initially attributed to amiodarone, which had been started for presumed new-onset atrial fibrillation and was promptly discontinued. Isoproterenol infusion was initiated for hemodynamic support and was associated with a transient acceleration of the ventricular response of atrial fibrillation. By hospital day 3, spontaneous conversion to sinus rhythm occurred.

Renal function deteriorated after contrast exposure, with the serum creatinine level increasing from 2.0 mg/dL on admission to a peak of 4.0 mg/dL on day 3, before gradually improving to baseline values at day 7.

**Figure 2:**
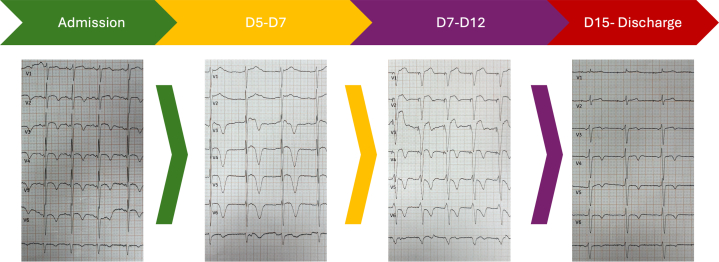
Temporal Evolution of Precordial Lead Electrocardiographic Changes Timeline illustrating the evolution of repolarization abnormalities in the precordial leads during hospitalization. At admission (day 0), the electrocardiogram demonstrates mild T-wave negativity in leads V_2_-V_6_, accompanied by subtle ST-segment elevation without an ischemic pattern and a normal QT interval. Between days 5 and 7, marked repolarization abnormalities develop, characterized by deeply inverted T waves in the precordial leads and severe QT prolongation (corrected QT interval up to 620 milliseconds). From days 7 to 12, during ventricular pacing from a temporary transvenous pacemaker, T-wave inversions become less pronounced, with partial shortening of the QT interval (approximately 480-500 milliseconds). By day 15, after permanent pacemaker implantation with atrial pacing, precordial T-wave inversions are minimal, and the QT interval has further shortened to approximately 440 milliseconds, reflecting progressive electrical recovery.

On day 5, despite the absence of any antiarrhythmic or bradycardic medical therapy, the patient again developed junctional rhythm accompanied by severe QT prolongation (up to 620 milliseconds) and diffuse T-wave inversion (Figure 2). In the meantime, the differential diagnosis of myocarditis was in the focus after the patient reported a self-limited gastrointestinal illness approximately 20 days before admission.

**Figure 3:**
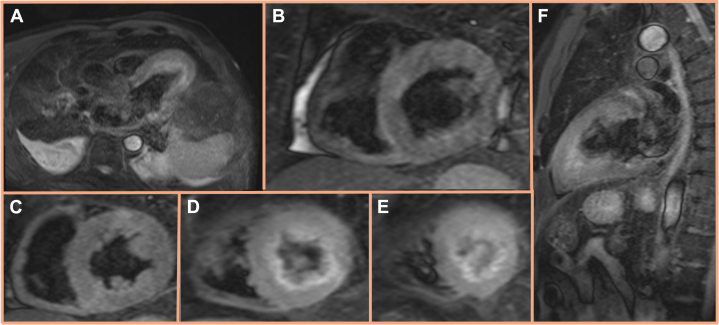
Cardiac Magnetic Resonance Findings Consistent With Stress Cardiomyopathy (A) T2-weighted short tau inversion recovery (STIR) short-axis view demonstrating increased myocardial signal intensity predominantly in the mid-to-apical left ventricular segments, consistent with myocardial edema extending beyond a single coronary territory. (B to E) T2-weighted STIR serial short-axis views from basal to apical segments demonstrating myocardial edema involving the mid and apical segments with apical predominance and relative basal sparing, extending beyond a single coronary territory, consistent with stress cardiomyopathy. (F) Late gadolinium enhancement phase-sensitive inversion recovery long-axis view demonstrating the absence of myocardial enhancement.

Cardiac magnetic resonance (CMR) performed on hospital day 7 demonstrated apical ballooning with akinesis of the apical and midventricular segments, myocardial edema in a distribution typical of stress cardiomyopathy, and no late gadolinium enhancement, making myocarditis and AIC unlikely (Figure 3, Video 2). Endomyocardial biopsy was therefore not performed given the CMR findings and the patient's hemodynamically stable clinical presentation, as the procedural risk was considered unjustified.

## Management

Initial management during admission focused on treatment of acute pulmonary edema and hypertensive crisis with intravenous diuretics and vasodilators, resulting in rapid clinical improvement. Rhythm control with amiodarone was initially pursued due to suspected new-onset atrial fibrillation and possible AIC but was discontinued after early development of profound bradyarrhythmia and QT prolongation (on day 1).

Isoproterenol infusion was used again, on day 5, for recurrent bradycardia, resulting in an accelerated junctional rhythm of approximately 80 beats/min, suggesting preserved atrioventricular conduction and indicating that the conduction disturbance was more likely localized to the sinus node rather than the atrioventricular node. After confirmation of stress cardiomyopathy on CMR, isoproterenol was discontinued because of concern for catecholamine-mediated myocardial injury, and a temporary transvenous pacemaker was implanted. Initial atrial pacing resulted in effective atrial capture with intact atrioventricular conduction, further confirming intact atrioventricular-node conduction but sinus node dysfunction (SND). For safety reasons, given concern for potential progression to atrioventricular conduction disturbances that have been described in TTS, the pacing lead was subsequently advanced to the right ventricle, and ventricular pacing was instituted.

Guideline-directed medical therapy for heart failure with reduced ejection fraction was initiated, including sacubitril/valsartan, dapagliflozin, and mineralocorticoid receptor antagonism. Low-dose intravenous β-blocker therapy (landiolol 1 μg/kg/min) was cautiously introduced under pacing support both for TTS management and because of significant QT prolongation. From day 8 onward, progressive shortening of the QT interval and improvement of repolarization abnormalities were observed, accompanied by gradual recovery of LV systolic function. β-Blocker therapy was discontinued on day 11 after QT interval improvement to reassess intrinsic sinus node function. By day 14, LV ejection fraction had normalized, whereas QT interval and T-wave abnormalities had substantially improved but not fully resolved (Figure 2). Before the decision for permanent pacing, the patient was observed under backup pacing at 30 beats/min, and atrial pacing was applied for 30 minutes to assess for sinus rhythm recovery. In addition, lower-limb movements and intravenous atropine were administered to evaluate chronotropic response, without evidence of sinus node activity; only junctional rhythm with a maximal rate of approximately 40 beats/min was documented. Despite prolonged observation (14 days) under temporary pacing support, sinus node function did not recover. After multidisciplinary evaluation and shared decision-making with the patient, a permanent dual-chamber pacemaker was implanted on day 15.

## Outcome and Follow-Up

**Figure 4:**
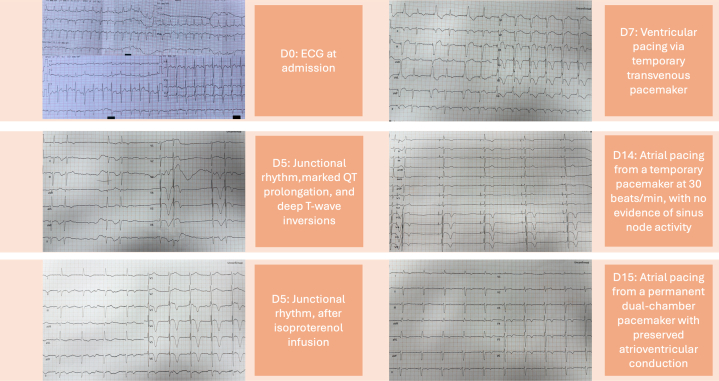
Serial Electrocardiograms Obtained During Hospitalization Demonstrate the Dynamic Evolution of Rhythm and Repolarization Abnormalities The admission electrocardiogram (ECG) shows atrial fibrillation with rapid ventricular response. During the acute phase of stress cardiomyopathy, profound sinus node dysfunction manifested as junctional rhythm with marked bradycardia, severe QT prolongation, and deep T-wave inversions. Despite progressive improvement of repolarization abnormalities and recovery of left ventricular systolic function, no sinus node activity was observed during prolonged observation under temporary pacing support, including periods of ventricular pacing at a low backup rate. Following permanent dual-chamber pacemaker implantation, atrial pacing with preserved atrioventricular conduction is demonstrated.

LV systolic function fully recovered within approximately 15 days, with an ejection fraction of 50% to 55% (Video 3). The QT interval normalized to approximately 440 milliseconds, and T-wave abnormalities markedly improved. Cardiac troponin I levels demonstrated a progressive decline from hospital day 3, measuring 101 pg/mL on day 5 and 51 pg/mL at discharge. B-type natriuretic peptide levels decreased to 400 pg/mL on hospital day 7, with no additional measurements obtained thereafter. Despite complete ventricular recovery, SND persisted (Figure 4).

The patient was discharged in stable condition with atrial pacing at 60 beats/min from a permanent dual-chamber pacemaker. At clinical reassessment 20 days later, electrocardiography again demonstrated atrial pacing, with no evidence of sinus rhythm recovery.

## Discussion

TTS is a reversible cardiomyopathy characterized by transient ventricular systolic dysfunction beyond a single coronary territory, often triggered by emotional or physical stress.^1^ LV recovery typically occurs within days to weeks; delayed recovery beyond 10 days has been reported and may be associated with a more complicated clinical course, with our patient recovering at 14 to 15 days.^2^^,^^3^ Although arrhythmias are frequent in acute TTS, clinically significant SND is rare, with sinoatrial block reported in approximately 1.3% and sick sinus syndrome mainly described in case reports.^4^

Only 2 prior reports have specifically described SND in TTS,^5^^,^^6^ both involving older patients with comorbidities or preexisting sinus node disease. In contrast, our comparatively younger patient had no prior conduction abnormality, making the occurrence and persistence of severe SND unexpected.

Arrhythmias in TTS range from atrial fibrillation to ventricular tachyarrhythmias and conduction disturbances.^7^ Although ventricular arrhythmias are often transient and related to QT prolongation, conduction abnormalities such as SND may persist beyond ventricular recovery.^4^^,^^7^^,^^8^ Proposed mechanisms include catecholamine excess, autonomic imbalance, microvascular dysfunction, and localized myocardial edema, potentially affecting the sinus node region.^4^^,^^7^^,^^8^

Pharmacologic management in TTS remains largely consensus-based rather than supported by randomized trials. In particular, the benefit of β-blockers remains unproven; ongoing randomized evaluation^9^ may clarify their role. Until such data are available, β-blockade should be considered empirically driven.

The role of cardiac implantable electronic devices in TTS remains debated. Available data suggest that permanent pacemaker implantation is more frequently required than implantable cardioverter-defibrillator therapy, reflecting the reversible nature of most ventricular arrhythmias.^4^^,^^8^^,^^10^ In our case, SND persisted despite complete LV recovery and the absence of chronotropic response after 14 days of observation, supporting permanent pacing. This case highlights that restoration of ventricular function does not necessarily imply recovery of intrinsic sinus node activity, underscoring the need for careful rhythm monitoring and individualized electrophysiological assessment.

## Conclusions

Stress cardiomyopathy may be complicated by persistent SND despite complete recovery of ventricular systolic function. Because of its rare presentation, careful rhythm monitoring and individualized treatment decisions are essential.

## Funding Support and Author Disclosures

The authors have reported that they have no relationships relevant to the contents of this paper to disclose.

## Appendix

Video 1 Transthoracic Echocardiography Performed at Admission Demonstrating Severely Reduced Left Ventricular Systolic Function With Diffuse Hypokinesis and an Estimated Ejection Fraction of Approximately 30% to 35%

Video 2 Cardiac Magnetic Resonance Performed on Hospital Day 7 Demonstrating Typical Apical Ballooning With Akinesis of the Apical and Midventricular Segments, Consistent With the Characteristic Pattern of Takotsubo Syndrome

Video 3 Follow-Up Transthoracic Echocardiography Obtained Before Discharge Demonstrating Marked Improvement of Left Ventricular Systolic Function With Normalization of Global Contractility and an Estimated Ejection Fraction of Approximately 50% to 55%
